# Adverse Mental Health Impact of the COVID-19 Lockdown in Individuals With Tourette Syndrome in Italy: An Online Survey

**DOI:** 10.3389/fpsyt.2020.583744

**Published:** 2020-11-30

**Authors:** Giulia Conte, Valentina Baglioni, Francesca Valente, Flavia Chiarotti, Francesco Cardona

**Affiliations:** ^1^Department of Human Neurosciences, Institute of Child and Adolescent Neuropsychiatry, Sapienza University of Rome, Rome, Italy; ^2^Center for Behavioral Sciences and Mental Health, Istituto Superiore di Sanità, Rome, Italy

**Keywords:** Tourette syndrome, COVID-19, pandemic, mental health, tics, children, adolescents

## Abstract

During the early stages of the coronavirus disease 2019 (COVID-19) pandemic in Italy, an online survey was launched via a local patient advocacy website to investigate mental health issues in children and adolescents with Tourette syndrome (TS). Respondents were parents, who were asked to report on their child's general health, tics, comorbidities/problems, pharmacological treatment/psychotherapy, symptom variations, and daily routine, as well as on their family's health and work experiences during the pandemic. Two hundred thirty-eight people participated in the survey, 203 females and 35 males. Our findings indicate that, in the time window of 4–6 weeks after the beginning of the COVID-19-related lockdown, 67% of individuals with TS developed a relevant worsening of the overall clinical condition as rated by their parents. An improvement or no variation of the clinical picture was reported in 20.5 and 6.7% of cases, respectively. Most worsened symptoms included tics, hyperactivity, rage attacks, obsessions/compulsions, and anxiety. Of the subjects experiencing a clinical worsening, the majority (51.76%) showed variations across two to five symptom domains. No association was found between symptom variation and family demographics or health and economic issues specifically related to the lockdown. The current COVID-19 pandemic is exerting a considerable impact on the mental health of young individuals with TS by worsening both tics and emotional and behavioral symptoms.

## Introduction

At the end of February 2020, the epicenter of the current coronavirus disease 2019 (COVID-19) pandemic shifted from China to Europe, with Italy being the first country to witness a massive peak in infections and fatalities (1, 2). In the past few months, the Italian government has put forward unprecedented measures to contain the outbreak, placing residents in a nationwide lockdown, and banning any movement inside the country. Such measures were ongoing until 4 May (3).

Each pandemic poses particular mental health risks in that it implies not only the spreading of a physical illness but also an overflow of anxiety and mass panic as possible reactions to health concerns. Moreover, common measures implemented by governments to restrain the outbreak, such as social distancing, isolation, and contact tracing policies, may further disrupt the collective sense of reality and order, worsening the emotional and psychological burden experienced by individuals (4). Previous research dealing with the mental health impact of pandemics has mainly focused on subjects put into isolation or quarantine, demonstrating psychiatric complications, such as anxiety and depression, in up to 40% of cases (5, 6). In the few studies regarding pediatric populations in similar conditions, externalizing and disruptive behaviors have also been frequently reported (7, 8).

Under such circumstances, those with a pre-existing mental health condition and especially children and adolescents with neuropsychiatric disorders appear as a particularly vulnerable population (9).

Tourette syndrome (TS) is a neurodevelopmental chronic condition defined by multiple motor and phonic tics (10). TS is a multifaceted disorder and is highly comorbid with conditions such as obsessive-compulsive disorder (OCD) and anxiety and attention deficit/hyperactivity disorder (ADHD) (11).

Although the underlying pathophysiological mechanisms is still unknown, a distinctive feature of TS is the great temporal variability of symptoms, both of tics and comorbidities (12). In TS patients, symptom expression is also systematically influenced by contextual factors (13) and is generally increased at home than in more social situations (14). Therefore, environmental factors such as the lockdown due to COVID-19 may significantly contribute in TS to concurrent symptom fluctuations, especially through mechanisms of setting modification and habit disruption, as resulting from mass school closures and stay-at-home orders.

In this study, we sought to provide an overview of the emotional and behavioral symptoms expressed by children and adolescents with TS in the early stages of the COVID-19 lockdown in Italy. We were also interested in determining whether symptom variations were associated with any specific sociodemographic variable or with health and economic issues developed during the pandemic within their families.

## Materials and Methods

### Data Source

For data collection, a brief online survey was developed by the Department of Human Neurosciences at Sapienza University of Rome and promoted by “Tourette Roma onlus,” a local patient advocacy association. The survey was launched on the association's website on 1 April 2020 (i.e., 3 weeks after the beginning of the general lockdown in Italy) and online records were collected until 14 April 2020). The study was conformed to the Declaration of Helsinki, and each submitted record was completely anonymous, therefore exempting the study from institutional Ethics Committee examination. The survey was directed to parents of individuals with TS and included different sections, with a total of 26 multiple-choice questions. Originally, the online form was developed for the parents of children and adolescents; however, a certain number of parents of adult patients also participated. Therefore, we decided to include the latter data in our analysis to offer a wider point of view on the investigated topic.

Participants were asked to report on their child's general health, tics, comorbidities/problems, variations of symptoms during the lockdown, presence and eventual changes in the pharmacological treatment/psychotherapy. Moreover, a survey section was dedicated to family demographics and impact of the pandemic on the child's daily activities as well as on family health and work experiences.

### Data Analysis

Data are presented as absolute and percent frequencies. Percent frequencies were calculated on the number of respondents to each item. The chi-square test was used to assess the effect of both participants' and offspring's variables on the variation of TS symptoms and quality of parent-child relationship. Participants' variables were as follows: age, gender, education, occupation, presence of domestic help before the pandemic (i.e., babysitter, grandparents/relatives looking after the child), geographical area of residence, living with partner, number of offspring and of family members, medical problems related to COVID-19, and economic changes related to the pandemic. Offspring's variables included the following: age, gender, comorbidities, pharmacological therapy and need of eventual adjustment during the lockdown phase, psychotherapy and its eventual variation due to lockdown, and time spent in school and other activities during the lockdown.

Because of the exploratory design of the study, we present the values of significance without correcting for multiple testing.

## Results

### Participants

Two hundred thirty-eight people participated in the survey. Of them, 203 (85.29%) were females and 35 (14.71%) males. The general rate of responses was 81%.

Table 1 displays geographic distribution, age, education level, employment status and family demographics of the participants. Participants living in Regions with medium rates of COVID-19 infections (i.e., number of confirmed cases/resident population, following the official data of the Italian Minister of Health updated as of 14 April 2020) were the most prevalent (46.64%), although highly impacted Regions were also largely represented (39.92%).

**Table 1:** Socio-demographic characteristics of participants.

	***n***	**%**
**Geographic distribution**		
Regions with high number of confirmed cases^*^ (Lombardy, Veneto, Emilia—Romagna, Piedmont, Trentino, Liguria, and Marche)	95	39.92
Regions with medium number of confirmed cases^*^ (Lazio, Tuscany, Campania, Abruzzo, Apulia, Friuli VG, Valle d'Aosta, Molise, and Umbria)	111	46.64
Regions with low number of confirmed cases^*^ (Calabria, Sicily, Sardinia, and Basilicata)	32	13.44
**Age**		
20–30 years	5	2.10
31–40 years	49	20.59
41–50 years	120	50.42
51–60 years	53	22.27
>61 years	11	4.62
**Education level**		
Primary	5	2.10
Lower secondary	36	15.13
Upper secondary	120	50.42
Bachelor or equivalent level	77	32.35
**Employment status**		
Not employed	17	7.14
Employee	124	52.10
Self-employed or freelancer	36	15.13
Homemaker	51	21.43
Retired	10	4.20
**Number of offspring**		
1	54	23.11
2	144	60.50
3	36	15.13
>3	3	1.26
**Number of people living at home (including relatives)**		
2	11	4.62
3	62	26.05
4	133	55.88
5	28	11.76
>5	4	1.68

* *Number of cases/resident population following the official data of the Italian Minister of Health*.

### Impact of the COVID-19 Epidemic on Family Health and Parents' Employment and Economic Status

About a quarter of the participants (*n* = 60 out of 230 who answered this question; 26.09%) have reported COVID-related health issues, such as hospitalization, illness or death among family members/friends, quarantine after contact with a confirmed case, or need for swabbing. Remarkably, about 32% of participants or their partners experienced a considerable reduction of the income due to unexpected lay-off or job uncertainties as a result of the pandemic, with 17% facing such consequence both themselves and their partners.

### Participants' Offspring With Tourette Syndrome

We gathered data on 223 participant's offspring with Tourette Syndrome.

Number of males was 188 (84.30%) and females 35 (15.70%), male-to-female ratio of 5.4–1.

Eighty-four subjects (37.67%) were in the age range between 6 and 11 years, 106 (47.53%) between 12 and 18 years, and 33 (14.80%) were older than 18 years.

Disease duration was of 1–2 years in 15 cases (6.73%), of 3–5 years in 78 (34.98%), of 6–10 years in 92 (41.25%), and >10 years in 38 (17.04%). TS diagnosis had been established for 1–2 years in 82 cases (36.77%), 3–5 years in 82 (36.77%), 6–10 years in 40 (17.94%), and more than 10 years in 19 (8.52%).

One hundred thirty-six (60.99%) subjects were medicated—of whom, 119 (53.36%) receiving medication for tics and 17 (7.62%) for other neuropsychiatric symptoms—while the remaining 87 (39.01%) were unmedicated at the time of the investigation.

One hundred thirteen (50.68%) subjects were receiving psychotherapy (any kind)—of whom, 66 (29.60%) for tics and 47 (21.08%) for other neuropsychiatric symptoms.

Table 2 shows the main reported comorbidities and which symptoms were rated as most impairing by parents during the lockdown period. Major pre-existing comorbidities of our sample were OCD, ADHD, anxiety, and rage attacks, with each disorder affecting about 50% of individuals. To possibly capture any other problem or symptom not otherwise specified by the survey checklist, an item “other problems” was included (Table 2), which needed a written specification. Symptoms recorded under such label included the following: oppositional behavior (*n* = 2), autism (*n* = 1), bipolar disorder (*n* = 1), non-suicidal self-injurious behaviors (*n* = 1), stuttering (*n* = 1), and bruxism (*n* = 1). Symptoms rated by parents as most impairing during the lockdown included mainly motor and phonic tics (22.84 and 14.21%, respectively), followed by rage attacks (16.75%), obsessions and compulsions (11.68%), and hyperactivity/inattention symptoms (9.64%).

**Table 2:** Reported comorbidities in the participants' offspring and symptoms rated as currently most impairing.

	**Present**		**Currently most impairing**	
	***n***	**%**	***n***	**%**
**Comorbid symptoms**				
Motor tics	N/A	N/A	45	22.84
Phonic tics	N/A	N/A	28	14.21
Obsessions/compulsions	106	56.08	23	11.68
Hyperactivity/inattention	103	54.50	19	9.64
Rage attacks	93	49.21	33	16.75
Anxiety	96	50.79	10	5.08
Depression	31	16.40	4	2.03
Panic	17	8.99	0	0
Learning problems	52	27.51	0	0
Sleep problems	53	28.04	5	2.54
Food problems	42	22.22	4	2.03
Other problems	24	12.70	12	6.09
None	35	15.69	14	7.11

### Changes in School and Daily Activities of Offspring During the Lockdown

One hundred eighty-nine subjects (87.10%) were students and participated in online learning at home. Along with online classes, 154 of them (70.97%) were also doing individual homework. Daily, the time spent in school activities was “ <1 h” in 43 cases (19.82%), “1 h” in 33 (15.21%), “2 h” in 48 (22.12%), “3 h” in 40 (18.43%), and “more than 3 h” in 53 (24.42%).

Apart from school activities, parents reported of their children spending time in the following: video games (n. 150; 69.12%), social media and chat (117; 53.92%), table or role games (64; 29.49%), playing an instrument or singing (38; 17.51%), household chores (60; 27.65%), painting (52; 23.96%), reading novels or comics (37; 17.05%), and other activities (63; 29.03%).

### Variations in Clinical Symptomatology and in Parent-Child Relationship During the Lockdown

The participants were asked to report variations of clinical symptoms in their offspring during the lockdown. The variations were expressed through a 5-point Likert scale (“much improved”, “improved”, “as usual”, “worsened” and “much worsened”).

A significant worsening of the overall clinical picture occurred in two-thirds of the subjects (67.18%), while 20.51 and 5.64% experienced, respectively, an improvement or a variation with no clear trend toward improvement/worsening and only 6.67% reported no variation at all (Table 3). Most worsened symptoms (Table 4), as rated by parents in over 25% of subjects, included tics, hyperactivity, rage attacks, obsessions/compulsions, and anxiety. Nineteen participants (9.55% of respondents) also reported of other problems, such as apathy, need for physical contact, stuttering, and argumentative/defiant behavior. Of the subjects experiencing a clinical worsening, the majority (51.76%) showed variations across two to five symptom domains (Table 4).

**Table 3:** Number and percentage of offspring with reported variation of symptoms during the lockdown.

	**6–11 years**	**12–18 years**	**>18 years**	**Total**
	***n***	***n***	***n***	***n* (%)**
No variation in any symptom	8	4	1	13 (6.67%)
Only improved symptoms (at least 1)	11	16	1	28 (14.36%)
Improved symptoms outnumber the worsened ones	5	4	3	12 (6.15%)
Improved symptoms equal the worsened ones	1	10	0	11 (5.64%)
Worsened symptoms outnumber the improved ones	18	13	4	35 (17.95%)
Only worsened symptoms (at least 1)	35	45	16	96 (49.23%)

**Table 4:** Type and number of “worsened” or “much worsened” symptoms during the lockdown according to age group.

	**6–11 years**	**12–18 years**	**>18 years**	**Total**
	***n***	***n***	***n***	***n* (%)**
**Type of symptom “worsened” or “much worsened”**				
Obsessions/Compulsions	20	25	11	56 (28.28%)
Hyperactivity	33	28	7	68 (34.34%)
Rage attacks	31	23	11	65 (32.82%)
Motor tics	33	41	10	84 (42.42%)
Phonic tics	29	36	7	72 (36.36%)
Anxiety	19	21	12	52 (26.26%)
Depression	8	12	8	28 (14.14%)
Sleep problems	16	23	8	47 (23.74%)
Panic	7	5	4	16 (8.08%)
Eating problems	11	20	7	38 (19.19%)
**Number of symptoms “worsened” or “much worsened”**				
0	19	20	2	41 (20.81%)
1	9	16	5	30 (15.23%)
2	15	13	3	31 (15.75%)
3	10	13	5	28 (14.21%)
4	12	15	3	30 (15.23%)
5	5	6	2	13 (6.59%)
6	7	4	3	14 (7.11%)
7	1	2	0	3 (1.52%)
8	2	2	0	4 (2.03%)
9	0	1	1	2 (1.01%)
10	0	0	1	1 (0.51%)

Finally, 111 respondents (55,78%) rated the quality of their parent-child relationship during the lockdown as being “as usual,” while it “improved” for 51 (25.63%), and “worsened” for 37 (18.59%).

### Associations

Contingency tables showed only few definite associations between the categorical variables listed in Methods and the variation either of symptoms of TS or of the quality of parent-child relationships during the lockdown.

In particular, a younger participant's age was associated to a worsening in the relationship with their child (33.33 vs. 17.82 vs. 7.69% in participants aged ≤ 40 years, 41–50 years, and >50 years, respectively; *p* = 0.0141). In addition, the quality of the parent-child relationship was associated to the presence of domestic helps before the pandemic, in that “improvement” and “worsening” of the relationship were respectively higher in the group of participants without and with domestic helps (improvement, 42.25%; worsening, 25.35%; *p* < 0.001). Finally, a shorter time dedicated to school activities during the lockdown was associated to a worsening of rage attacks, obsession, and compulsions and of the overall clinical symptomatology (*p* = 0.0305, *p* = 0.0309, and *p* = 0.0415, respectively).

## Discussion

Based on parent reports among a national sample, the present study highlights the adverse impact of the COVID-19 pandemic on mental health of individuals with TS.

Although limited by its cross-sectional design, our study points out an important worsening of the overall clinical condition in the time window of 4–6 weeks after the beginning of the lockdown. Specifically, over 67% of parents reported worsening of TS symptoms, while only 20.5 and 6.7%, respectively observed improvement or no variation of the clinical picture. Such large and definite trend toward worsening in our sample seems unlikely related to the polyphasic course of TS, with the typical fluctuations in the severity of tics and associated symptoms. Furthermore, our data do not support any association between variation of symptoms and family health and economic issues related to the pandemic. The latter aspect might reflect either a lack of statistical power of our data or the involvement of other factors contributing to adverse mental health outcomes in our cohort, such as the condition created by the lockdown itself and the inherent daily life disruption.

Tics, especially motor ones, were rated in up to 42% of cases as the most worsened symptoms. As a general rule, individuals with TS tend to tic less when they are engaged in social situations (e.g., school or work settings) and more when they are relaxed or with family (14, 15). In line with this evidence, our data suggest that the prolonged stay-at-home condition during the lockdown may have contributed to tic exacerbation.

Importantly, the specific population here examined was part of a larger community experiencing a collective stressful life event such as the COVID-19 pandemic. Psychosocial stress and adverse life events have been largely implicated not only in tic frequency and severity (16) but also in the worsening of comorbid depression and anxiety in TS (17). Accordingly, we observed an increase of anxiety and obsessive-compulsive symptoms (OCS) in 26 and 28% of our sample. However, our findings indicate that anxious-depressive symptoms were outreached by externalizing and behavioral problems. Indeed, outbursts of anger (“rage attacks”) and problems related to hyperactivity/impulsivity, regarded almost 32–34% of the subjects and especially those in the younger age groups (6–11 and 12–18 years old). Such different rates of externalizing and internalizing problems parallel the findings from previous studies on hospitalized patients put into isolation [for a review (18)], showing that, while adults were more at risk for depression and general anxiety disorder, children presented more often with externalizing and disruptive behaviors. Of note, if compulsions and anxiety may represent dysfunctional coping strategies aimed at gaining control over a situation of unexpected threat (19), anger, and irritability have also been associated in children and youths with biased attention toward threatening information (20–22). Against this background, our findings support that the stressful conditions established by the lockdown, such as habit and social contact disruptions, may have acted as threat cues for patients, exacerbating dysfunctional behavioral and emotional responses.

Adding to this, we found an inverse relation between time dedicated to school activities during lockdown and the overall clinical symptomatology (i.e., shorter time dedicated to schoolwork was significantly related to greater worsening of symptoms, most notably rage attacks and OCS). Intuitively, subjects facing greater symptom exacerbation during the lockdown may have been more prone to concentration issues. Also, a reduced time for sport and physical activity, given their positive impact on well-being (23) and learning achievements in children (24), may have influenced the amount of time dedicated to remote learning and the general mental health of our sample. Taken together, the worsened clinical condition and the reduced possibility of physical exercise may have impacted on the time spent in schoolwork by patients in our cohort, with a shift toward activities such as video games and social networking, as already reported in a population-based study on Middle Eastern Respiratory Syndrome (MERS) (25).

Finally, the present study outlines no negative effect of prolonged stay at home on parent-child relationships, which are referred as stable or even improved in the large majority of cases. In this regard, the only variable correlating to worsening was the presence of extended family help before the pandemic (i.e., grandparents/relatives looking after the child), which was presumably disrupted due to lockdown requirements.

Our study has several limitations, although some are common to the survey methodology. The choice of a digital survey was motivated by the need to rapidly reach out to a greater group of individuals living in areas of the country that were differently affected by the pandemic. As for surveys in general, our study has been prone to response biases which affect accuracy and consistency of the results. Nevertheless, to partly control for random answers and increase responders' reliability, we launched the survey through an advocacy group website, thus attempting to gather data from individuals who were familial with and interested in the topic of tic disorders. Given such demographic aspects, referral biases were also possible (i.e., a higher participation in the survey by those living the more stressful situations). However, our data revealed a considerable number of cases reported as stable or even improved. This issue also suggests the possibility of a mitigation effect played by the lockdown on tics and related symptoms in a specific subgroup of individuals. Whether a determinant or a consequence of negative social interactions, many children with tics experience high social anxiety (26, 27). Thus, reduction of societal stress due to limited social exposure may have been at play in part of the variance observed.

Finally, the cross-sectional and anonymous nature of our data has hindered the possibility of a follow-up. Future studies on longitudinal cohorts would clearly add deeper insight on long-term mental health impact of the current pandemic in selected subpopulations.

Taking into account these limitations, our study highlights that, for a substantial percentage of patients with TS, the lockdown imposed by the COVID-19 pandemic has been an extremely difficult period characterized by the worsening of the overall clinical condition. Remarkably, though based only on qualitative data, the increase of behavioral and emotional symptoms in our sample seems to parallel the increase in tic severity, which might be interpreted as a sign of the interrelatedness of symptoms in the broader TS spectrum.

There is a compelling need to carefully observe how this epochal event may impact on mental health and natural course of TS in the long term. In view of possible future lockdowns and prolonged stay-at-home orders, health authorities and clinicians should be aware of the risks for mental health in vulnerable populations, in order to ensure appropriate healthcare without undermining public health needs.

## Data Availability Statement

The raw data supporting the conclusions of this article will be made available by the authors, without undue reservation.

## Ethics Statement

Ethical review and approval was not required for the study on human participants in accordance with the local legislation and institutional requirements. Written informed consent to participate in this study was provided by the participants' legal guardian/next of kin.

## Author Contributions

GC and VB contributed to the conception, design of the study, and wrote the first draft of the manuscript. GC, VB, and FV organized the database. FCh performed the statistical analysis. FCa contributed to the conception, design of the study, and revised the manuscript. All authors contributed to manuscript revision, read, and approved the submitted version.

## Conflict of Interest

The authors declare that the research was conducted in the absence of any commercial or financial relationships that could be construed as a potential conflict of interest.
